# Potential Photoprotective Effect of Dietary Corn Silk Extract on Ultraviolet B-Induced Skin Damage

**DOI:** 10.3390/molecules24142587

**Published:** 2019-07-16

**Authors:** Yeon-hee Kim, Amy Cho, Sang-Ah Kwon, Minju Kim, Mina Song, Hye won Han, Eun-Ji Shin, Eunju Park, Seung-Min Lee

**Affiliations:** 1 Department of Food and Nutrition, BK21 PLUS Project, College of Human Ecology, Yonsei University, Seoul 03722, Korea; 2 Department of Food and Nutritional Science, Kyungnam University, Changwon 51767, Korea

**Keywords:** corn silk extract, photoaging, ultraviolet B, NF-κB, antioxidant, inflammation

## Abstract

Ultraviolet B (UVB) irradiation causes adverse effects on the skin. Corn silk contains flavonoids and other bioactive compounds and antioxidants, which may prevent skin photoaging through antioxidant and anti-inflammatory effects. We aimed to investigate the potential photoprotective effects of dietary corn silk on UVB-induced skin damage in mice and the mechanisms behind these effects on human skin cells. Oral administration of corn silk water extract (CS) (2 or 4 g/kg/day) for 19 weeks decreased epidermal thickness, wrinkle formation, and positive staining for PCNA, Ki67, and 8-OHdG, and increased collagen staining in UVB-irradiated SKH-1 hairless mice compared with controls. The pro-inflammatory NF-κB target genes (IL-1β, iNOS, and COX-2) and MMP-9 expressions were lower in the CS groups, and TGF-β/Smad signaling increased. Low skin lipid peroxidation and blood DNA oxidation levels and high blood glutathione were detected. Antioxidant transcription factor Nrf2-related catalase and SOD1 proteins and glutaredoxin mRNA levels increased. The results of CS extract treatment and UVB irradiation in HaCaT cells showed the same results in Nrf2 and NF-κB target genes. An LC-MS/MS analysis showed that the CS extract contained potential antioxidants, which might have contributed to its anti-photoaging effects in tissues and cells. CS extract may reduce UVB-induced skin damage through antioxidant and anti-inflammatory mechanisms.

## 1. Introduction

Skin, the largest organ in the human body, acts as a barrier against external pollutants [1]. Skin tissue is constantly exposed to solar ultraviolet (UV) radiation [2]. When the antioxidant defense system is damaged due to ultraviolet B (UVB) exposure of the skin, excessive reactive oxygen species (ROS) are generated at the irradiated sites [3], which results in various changes, including the oxidation of cellular components, DNA mutation, inflammation, and the activation of specific enzymes that degrade the extracellular matrix [4]. These changes can lead to unrestrained cell proliferation [5] and carcinogenesis [3]. Chronic exposure to UV leads to photoaging and even skin cancer [2]. Skin UVB-induced photoaging is associated with distinct clinical features, such as wrinkling and thickening of the epidermis, which is a protective layer of the skin [5,6]. There are also related histological characteristics such as disorganization of collagen in the dermis, which lies below the epidermis, fragmentation, and dispersion [7].

The antioxidant system is thought to protect against cellular damage from UV-induced oxidative stress through the scavenging of ROS or their byproducts [5]. Carotenoids, polyphenols, and vitamins E and C have been shown to act as photo-protective barriers by quenching ROS [8]. The oral administration of natural product extracts, such as French maritime pine (*Pinus pinaster*) bark, Ginkgo biloba [6], green tea (*Camellia sinensis*), and grape seed extracts [8], has been shown to prevent UV-induced skin damage. Thus, the consumption of herbal substances that are rich in antioxidant components, such as polyphenols, might offer protective effects against UVB damage [1].

Corn silk (CS, *Zea mays L*.) has been consumed as a herbal medicine in Korea [9], China, United States, and France for millennia [10]. In folk medicine, it is used for the treatment of cystitis, edema, diabetes mellitus, and prostatitis [10]. CS contains an abundance of phenolic compounds, such as flavonoids (maysin, apigmaysin, luteolin) [11,12], anthocyanins (cyanidin, peonidin) [13], chlorogenic acid, and other biologically active substances, such saponins and allantoin [11,14]. The antioxidant capacity of CS was first reported by Zoran et al., who showed a positive correlation between the polyphenol content and antioxidant activity in aqueous acetone CS extract in vitro [15]. Radical scavenging activity and iron chelating activity of CS extracts were proven in test tube experiments [16,17]. The antioxidant capacities of the CS extract have been implicated in human breast cancer cells [18], human neuroblastoma cells [19], clonal rat pancreatic β-cells [20], and CSP2, a polysaccharide isolated from the CS extract [21]. In animal experiments, ad libitum drinking of CS extract for 28 days in healthy albino mice elevated antioxidant enzyme activities and increased the content of reduced glutathione in the kidney [22]. In another animal study, dietary CS extract rescued a high salt diet-induced reduction in glutathione peroxidase [23] and alleviated radiation-induced oxidative stress in mice [24]. In addition, oral ingestion of CS-extracted flavonoids showed antioxidant effects against oxidative stress under exhaustive exercise [14] and under streptozotocin-induced diabetes [25] in mice.

The effects of CS extracts on UVB-induced damage have not been extensively studied. There were only several cell line experiments using CS constituents [26,27]. Trans-zeatin, purified from corn silk, inhibited UVB-induced MMP-1 expression in skin fibroblasts [26]. Luteolin, another flavonoid found in CS, exerted anticancer effects on UVB-irradiated mouse epidermal cells by suppressing cyclooxygenase (COX) expression and NF-κB activity [27]. The role of dietary CS extract on UVB-damaged skin and its antioxidant mechanism are still not clear. In our study, we aimed to demonstrate the effect of CS extract on preventing UVB-induced skin damage in mice and further confirm the molecular mechanisms underlying this effect in human skin cells. We hypothesized that the CS extract might contribute to its photoprotective effect through antioxidant and anti-inflammatory pathways. We investigated the effects of the oral administration or treatment of CS extract on UVB-induced damage in SKH-1 hairless mice and human skin HaCaT cells.

## 2. Results

### 2.1. DPPH and ABTS Antioxidant Capacities of CS Extract

**Figure 1:**
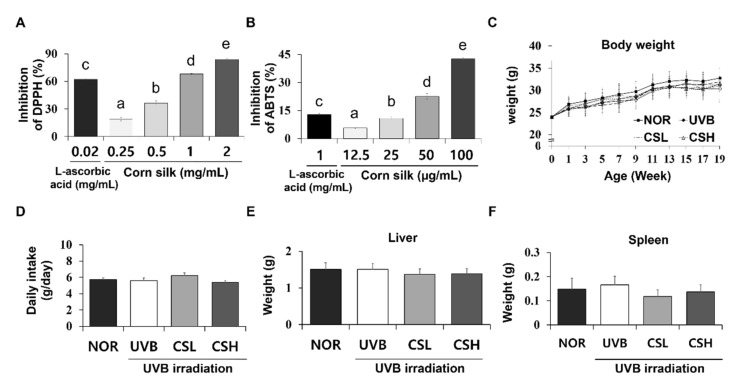
DPPH and ABTS antioxidative assay of corn silk (CS) extracts and the general details of SKH-1 mice during the experiment. Antioxidative effects of CS extracts were determined by DPPH (**A**) and ABTS radical scavenging activity (**B**). For the assays, CS extracts at concentrations of 1.25, 2.5, 5, 10 mg/mL and DPPH or ABTS solution were mixed at a ratio of 1:4 and 1:99. Body weight (**C**), food intake (**D**), liver weight (**E**), and spleen weight (**F**) of the mice were not significantly different across all groups (n = 8~10 per group) including normal control group (NOR), UVB-irradiated group (UVB), UVB-irradiated and low CS- (2 g/kg/day) treated group (CSL), and UVB-irradiated and high CS- (4 g/kg/day) treated group (CSH). Values are mean ± SD. The results were analyzed by one-way analysis of variance (ANOVA) followed by Duncan’s post-hoc test. Different lowercase letters over bars (a, b, c, d, e) represent significant statistical differences (*p* < 0.05).

DPPH (2,2-diphenyl-1-picryl-hydrazyl-hydrate) radical scavenging activities of CS extract varied from 18.99 to 83.92% (Figure 1A). ABTS (2,2’-azino-bis(3-ethylbenzothiazoline-6-sulphonic acid)) assay values ranged from 4.74 to 42.75% (Figure 1B). The half maximal inhibitory concentration (IC_50_) of CS extract and the standard compound, L-ascorbic acid, in relation to DPPH and ABTS radical scavenging activities (Table 1).

### 2.2. Food Intake, Body Weight, and Organ Weights of Animals

Body weights of the mice were not significantly different between groups during the study period (Figure 1C). Mean daily food intake was also similar between groups (Figure 1D). Mouse liver and spleen weights were not significantly different between the groups at sacrifice (Figure 1E,F).

### 2.3. Effects of CS Extract on Skinfold, Epidermal Thickness, and Wrinkle Formation in UVB-Irradiated Mice

**Figure 2:**
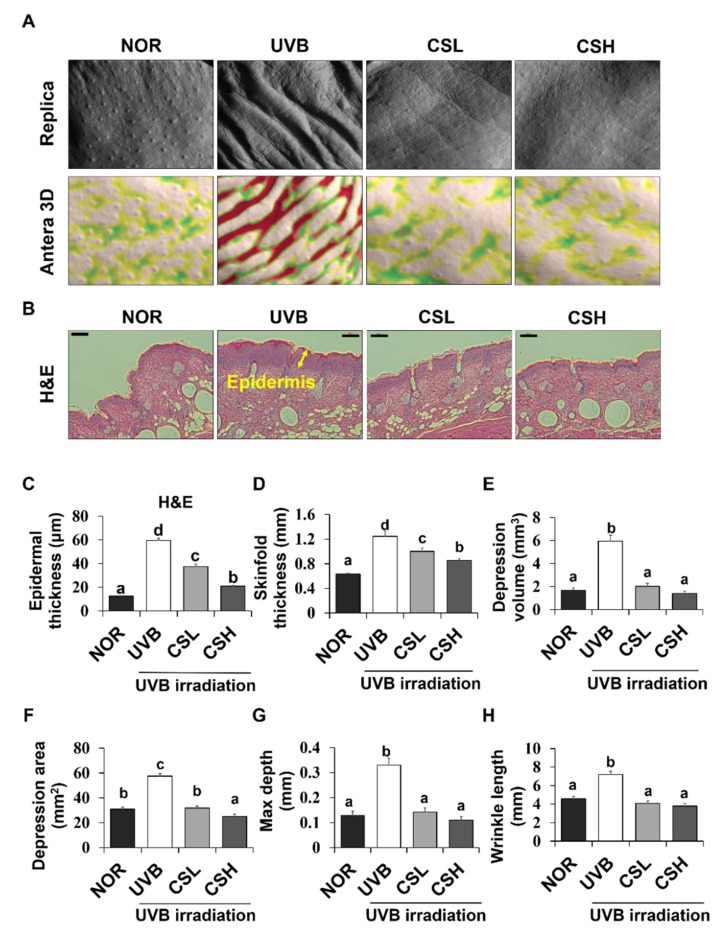
Effects of corn silk (CS) extract on UVB-induced wrinkle formation in the dorsal skin of SKH-1 mice at the end of the study (week 19). Photographs of the replica, replica analysis, and the backs of the mice (**A**), Hematoxylin and eosin-stained sections (original magnification 100×) (**B**), epidermal thickness (**C**), skinfold thickness (**D**), mean of skin wrinkle depression volume (**E**), mean depression area (**F**), maximum depth (**G**), and wrinkle length (**H**) are presented for normal group (NOR), UVB-irradiated group (UVB), UVB-irradiated and low CS- (2 g/kg/day) treated group (CSL), UVB-irradiated and high (4 g/kg/day) CS-treated group (CSH). Values are mean ± SD. The results were analyzed by one-way analysis of variance (ANOVA) followed by Duncan’s post-hoc test. Means with different lowercase letters (a, b, c, d) represent statistically significant differences (*p* < 0.05). Bars with the same letters are not significantly different.

The ANTERA 3D^®^ images of skin replicas of animal groups are presented in Figure 2A. UVB irradiation increased the epidermal thickness of the dorsal skin and the thickness of the skinfold compared with the NOR group (Figure 2B–D). However, administration of the CS extract significantly reduced the epidermal and skinfold thickness in the CSL and CSH groups compared with the UVB group (Figure 2B–D). UVB irradiation increased the values of all parameters related to wrinkle formation, including the volume of depression, the affected area of depression, the maximum valley depth, and the average wrinkle length in comparison to the NOR (Figure 2E–H). These values were significantly decreased in the CSL and CSH groups in comparison to the UVB group (Figure 2E–H).

### 2.4. Effect of CS Extract on Epidermal Expression Levels of PCNA and Ki67 in UVB-Irradiated Mice

**Figure 3:**
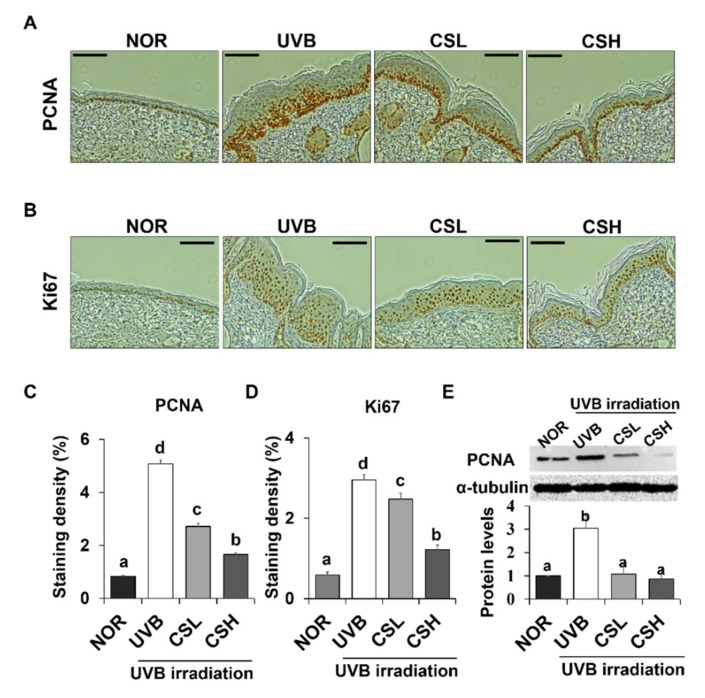
Effect of corn silk (CS) extract on the expression of PCNA and Ki67 in UVB-irradiated hairless mice skin. Representative images of immunohistochemical staining of PCNA (**A**) and Ki-67 (**B**) are shown. Immunostaining of each gene is depicted as brown areas at the original magnification of ×200. PCNA (**C**) and Ki-67 (**D**) were quantified using ImageJ software. Representative Western blot image and protein levels of PCNA (**E**) in mouse skin tissue are shown (repeated five times). Values are mean ± SE of the percentages of positive nuclear staining in the skin tissue or protein levels. The results were analyzed by one-way ANOVA with Duncan’s post-hoc test. Bars accompanying different lowercase letters (a, b, c, d) represent statistically significant differences (*p* < 0.05), whereas the same letters represent no significant difference. Group abbreviations: Normal group (NOR), UVB-irradiated group (UVB), UVB-irradiated and low CS- (2 g/kg/day) treated group (CSL), UVB-irradiated and high CS- (4 g/kg/day) treated group (CSH).

The cell proliferation levels in the dorsal skin sections of the animal groups were examined by the expression levels of proliferation marker genes PCNA (proliferating cell nuclear antigen) and Ki67 (Figure 3A–D). PCNA- and Ki67-positive cells were localized to the stratum basale (basal layer) between the epidermis and the dermis in the NOR whereas positive staining for PCNA and Ki67 were detected throughout several layers of the epidermis in the UVB group (Figure 3A,B). The positive staining levels of PCNA and Ki67 were significantly decreased in CSL and CSH groups compared with the UVB group (Figure 3C,D). Greatest reductions in the staining levels were detected in the CSH group (Figure 3C,D). The CS extract significantly rescued the UVB-induced increase in PCNA protein levels in mouse dorsal skin tissue (Figure 3E). These data indicated that the CS lessened the UVB-mediated epidermal cell proliferation.

### 2.5. Effect of CS Extract on Skin Collagen Fiber Content in UVB-Irradiated Mice

**Figure 4:**
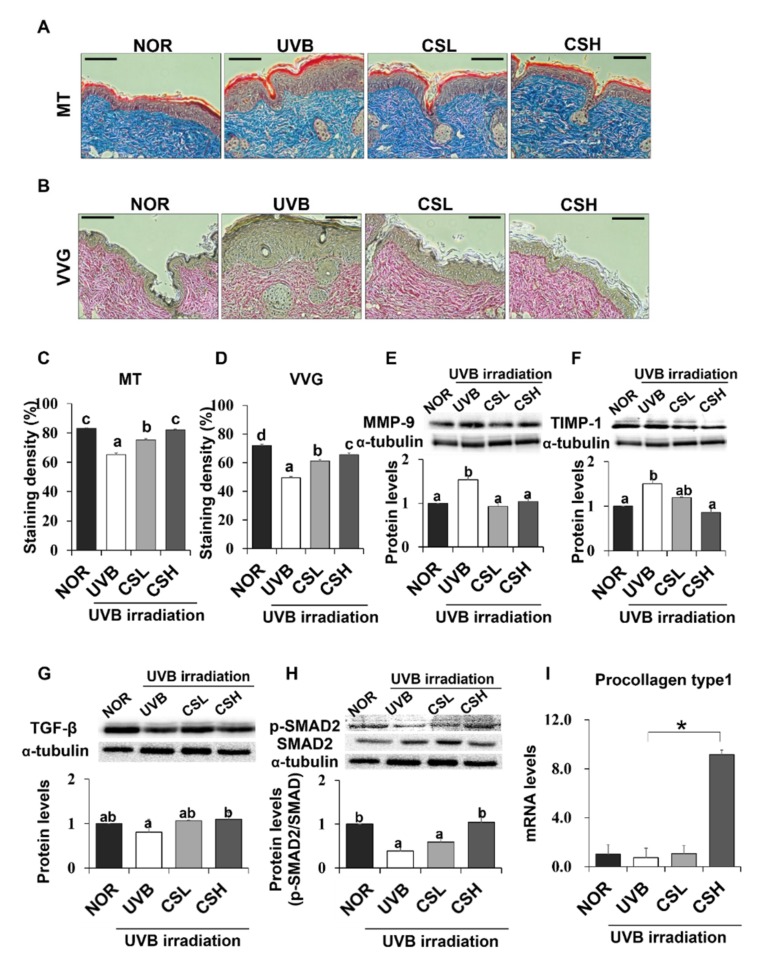
Effect of corn silk (CS) extract on collagen fiber content in UVB-photoaged mouse skin. Collagen fibers were stained with Masson’s trichrome (MT) (**A**) and Verhoeffe Van Gieson (VVG) (**B**). Representative histological images of collagen in mouse skin tissue are presented at an original magnification of ×200. Collagen staining by MT and VVG appears blue and red, respectively. Staining density of MT (**C**) and VVG (**D**) are shown, respectively. Western blotting detected MMP-9 (**E**), TIMP-1 (**F**), TGF-β (**G**), p-SMAD2/SMAD2 (**H**), and α-tubulin expression levels in the UVB-irradiated dorsal skin protein extract of hairless mouse by using specific antibodies for each protein. Blot image is a representation of three individual experiments. The blots were quantified using ImageJ software and the signal intensities were normalized to the value of α-tubulin, except for p-SMAD2, which was normalized to the expression of SMAD2. The mRNA expression of procollagen type 1 was assessed by q-PCR analysis and was normalized to the intensity value of GAPDH, quantified using ImageJ (**I**). Values are mean ± SE. Mean values not assigned with the same letter (a, b, c, d) are significantly different, analyzed by ANOVA (*p* < 0.05). Group abbreviations: Normal group (NOR), UVB-irradiated group (UVB), UVB-irradiated and low (2 g/kg/day) CS-treated group (CSL), UVB-irradiated and high (4 g/kg/day) CS-treated group (CSH).

Skin connective tissue levels were assessed by MT and VVG staining, which detect collagen fibers (Figure 4A,B). Compared with the NOR group, the UVB group showed lower staining levels in both MT and VVG (Figure 4C,D). The CSL and CSH groups partly reverted the UVB-induced loss of the staining density and the CSH showed the strongest staining compared to other groups in both MT and VVG (Figure 4C,D). Next, protein expression levels of matrix metalloproteinase-9 (MMP-9) and Tissue inhibitors of metalloproteinases (TIMP) which regulate extracellular matrix, were examined (Figure 4E,F). Protein levels of MMP-9 and TIMP-1 in the skin were increased by UVB irradiation compared with the NOR group, but CS treatment decreased MMP-9 at both low and high doses and reduced TIMP-1 levels at the high dose (Figure 4E,F). Activation of TGF-β and Smad2/3 signaling pathway was investigated in terms of collagen production [7]. The protein levels of TGF-β were not different between the UVB and the NOR groups, but there were modest elevations of TGF-β only in the CSL and CSH groups compared with the UVB group (Figure 4G). The UVB irradiation lowered phosphorylation levels of Smad2/3 compared with the NOR group, but this reduction was significantly reverted in the CSH while not in the CSL group (Figure 4H). Procollagen type 1 levels significantly increased in the CSH than in the UVB group (Figure 4I). These results suggested that UVB-diminished TGF-β and Smad2/3 signaling pathway were re-activated in the CSH, and possibly in the CSL.

### 2.6. Effect of CS Extract on Oxidative Stress and Skin Antioxidation Genes

**Figure 5:**
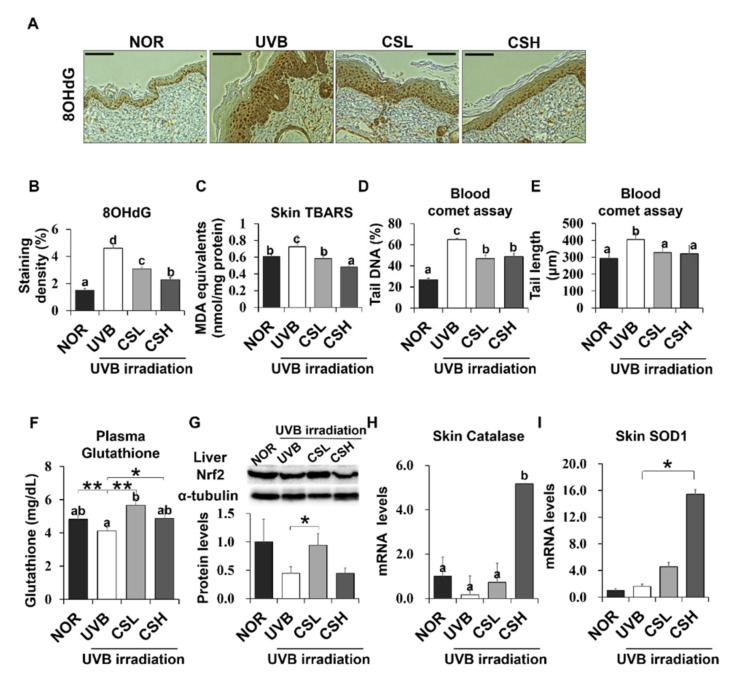
Effect of Corn Silk (CS) extract on antioxidant gene expressions in response to UVB-induced oxidative stress in mouse skin and liver. The UVB-induced formation of DNA/RNA damage marker, 8OHdG, was detected by immunohistochemistry. Representative images (**A**) and quantification (**B**) of the staining are shown. MDA in the skin tissue was measured by the TBARS assay (**C**). Oxidative stress in the blood was determined by the leukocyte comet assay and plasma glutathione concentration. DNA damage was detected by the tail DNA (%) (**D**) and tail length (μm) (**E**). Plasma glutathione (GSH) concentrations were compared (**F**). Nrf2 protein levels were assessed by Western blot and a representative image of the blot is shown (**G**). The mRNA expression of catalase (**H**) and SOD1 (**I**) are shown as assessed by q-PCR. Values are mean ± SE. Values with different letters (a, b, c, d) indicate statistical significance (*p* < 0.05), as analyzed by one-way ANOVA. * *p* < 0.05, ** *p* < 0.01, student t-test. Abbreviations: Normal group (NOR), UVB-irradiated group (UVB), UVB-irradiated and low (2 g/kg/day) CS-treated group (CSL), UVB-irradiated and high (4 g/kg/day) CS-treated group (CSH).

To investigate the effects of CS on UVB-induced oxidative stress, skin and blood oxidative stress-related markers were examined. The UVB group showed significant positive staining of 8-OHdG, an oxidative DNA damage marker, in the skin epidermis compared to the NOR group (Figure 5A,B). However, CS treatment reduced 8-OHdG levels, with a greater reduction in the CSH group (Figure 5A,B). In addition, lipid peroxidation products measured by Thiobarbituric acid reactive substances (TBARS) in the skin were significantly decreased in the CSH group (Figure 5C). UVB also increased the percentage of tail DNA and tail length of PBMC cells, indicating more DNA damage compared with NOR (Figure 5D,E). However, the tail DNA and tail length of PBMC cells were reduced in the CSL and CSH groups (Figure 5D,E). Plasma GSH, an antioxidant molecule, was slightly decreased in the UVB group compared with that in the NOR group (*p* = 0.041), but significantly increased in CSL (Figure 5F). These results indicated that the CS extract decreased UVB-induced skin and blood oxidative damage. 

UVB irradiation decreased Nrf2 protein expression in liver tissue, but CSL significantly recovered the UVB-induced loss of Nrf2 levels (Figure 5H). In addition, catalase and SOD1 expression significantly increased in skin tissue CSH compared with the UVB group (Figure 5H, I). As a result, CS reduced UVB-induced oxidative stress in the mouse skin and liver tissues.

### 2.7. Effect of CS Extract on Skin Inflammatory Gene Expression in UVB-Irradiated Mice

**Figure 6:**
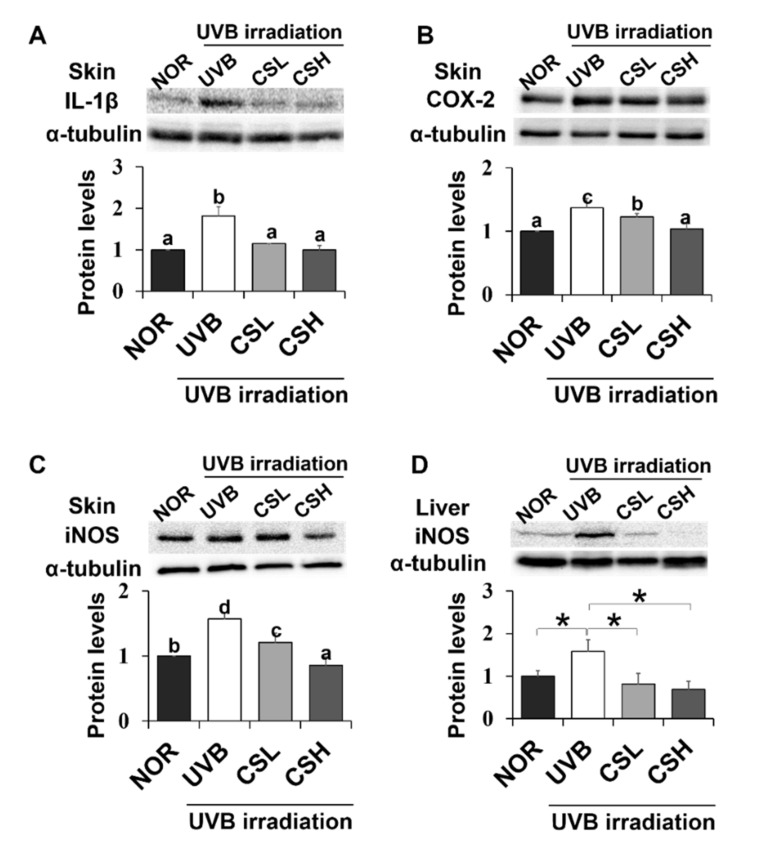
Effect of corn silk (CS) extract on inflammatory gene expressions in UVB-irradiated mice skin and liver. Protein expressions of IL-1β (**A**), COX-2 (**B**), and iNOS (**C**) in mouse skin, and iNOS in mouse liver (**D**) were assessed by Western blot analysis and their representative images of multiple independent experiments (*n* = 3) are presented. Protein results of iNOS in mouse liver is shown in (**F**) and its representative blot image is shown in (**E**). Results are expressed as mean ± SE. Mean values sharing different letters (a, b, c, d) over bars are significantly different (*p* < 0.05), as analyzed by ANOVA. * *p* < 0.05, student t-test.

Expression levels of NF-κB target inflammatory genes including iNOS, IL-1β, and COX-2 in UVB-irradiated mouse skin and liver tissue were examined (Figure 6A–D). In mouse skin, iNOS, IL-1β, and COX-2 levels increased in the UVB group compared with the NOR group and decreased in the CS groups compared with the UVB group (Figure 6A–C). In mouse liver, iNOS was decreased in CSL and CSH compared with the UVB group (Figure 6D). Therefore, CS decreased levels of inflammatory gene expression in UVB-irradiated skin and liver tissues.

### 2.8. Effect of CS Extract on UVB-Irradiated Human Keratinocytes

**Figure 7:**
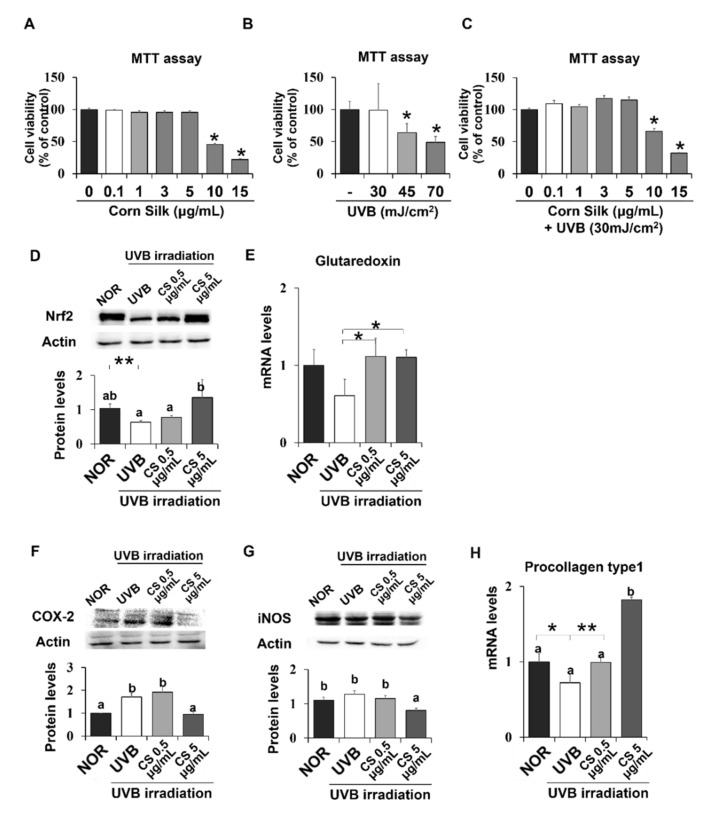
Viability of CS- or UVB treated HaCaT cells and CS effect on antioxidant and anti-inflammatory gene expressions in HaCaT cells. MTT assays showed the viability of HaCaT cells after exposure to either CS extract (**A**), only UVB (**B**), or both UVB and CS extract treatment (**C**). Protein levels of Nrf2 (**D**) were analyzed by Western blot and the representative images of the blot (three repetitions) are shown. Protein levels of COX-2 (**F**) and iNOS (**G**) were analyzed by Western blot and the blot images represent three repetitions. mRNA levels of Glutaredoxin (**E**), a target of Nrf2, and procollagen type 1 (**H**) was analyzed by qPCR and normalized to GAPDH. Results are mean ± SE. Different lowercase letters (a, b) represent statistical difference, as analyzed by ANOVA (*p* < 0.05). * *p* < 0.05, ** *p* < 0.01, student t-test.

Additional experiments were carried out on the human HaCaT keratinocyte cell line to further examine the mechanism of the CS extract’s photoprotective effect on skin. First, MTT assay was performed to investigate the cytotoxic effect of UVB and CS extract on HaCaT cells. A 24-h treatment of CS extract did not show cytotoxicity at concentrations up to 5 μg/mL (Figure 7A). UVB irradiation above 30 mJ/cm^2^ significantly inhibited cell growth in a concentration-dependent manner (Figure 7B). Therefore, the UVB dose for cell experiments was set as 30 mJ/cm^2^. In accordance with these results, CS treatment on 30 mJ/cm^2^ UVB-irradiated cells did not show cytotoxicity up to 5 μg/mL (Figure 7C). Based on this data, 5 μg/mL was defined as the higher concentration (CSH), and a ten-fold dilution of 0.5 μg/mL was determined as the lower concentration (CSL).

Expression levels of Nrf2 were confirmed in human skin cells, which showed similar positive effects of CS extract as did in mice. Nrf2 protein level was significantly reduced by UVB irradiation but recovered by CS treatment at 5 μg/mL (Figure 7D). Nrf2 target gene, glutaredoxin, showed similar regulation by CS treatment at both 0.5 and 5 μg/mL (Figure 7E). NF-κB target inflammatory genes, including COX-2 and iNOS, were also examined in UVB-irradiated epithelial HaCaT cells. COX-2 and iNOS were significantly decreased in the UVB group treated with CS 5 ug/mL compared with the non-CS treated UVB group (Figure 7F,G). Procollagen type 1 mRNA level was significantly improved in both doses of CS extract compared with the UVB group (Figure 7H). Overall, the CS extract improved Nrf2 signaling and alleviated inflammatory gene expression levels in UVB-irradiated HaCaT cells.

### 2.9. Metabolite Identification in CS Using LC-MS/MS

LC-MS/MS analysis revealed putative detection of 6083 peaks in the CS extract, and the top 100 peaks in the order of peak intensities were listed in Supplementary Materials (Table S1). The peak numbers below in parentheses indicate the order of the metabolites detected in the CS extract from highest to lowest peak intensities. Out of the top 100 peaks in the CS extract, the top 30 metabolites were further identified whether they exhibit antioxidant and/or anti-inflammatory effects based on previous studies. Indole (1^st^ peak), 3-(3-hydroxyphenyl)propanoic acid (2^nd^ peak), and proline betaine (7th peak) were revealed as the three most abundant metabolites in the CS extract that exhibit antioxidant and/or anti-inflammatory effects (Table S1). This suggested that the CS contained antioxidant compounds might have contributed to the antioxidant effect in skin protection. In mice, skin metabolites were compared between NOR, UVB, CSL and CSH groups (Figure S1A). Five hundred eight metabolites were altered in the CS groups compared to the UVB group (Figure S1B). Metabolites found in the CS extract were also detected in among the 161 up-regulated metabolites in the skin of CS groups compared with the UVB group. These included proline betaine (7th peak), L-proline (in the form of L-phenylalanyl-L-proline, 11th peak), L-phenylalanine (in the form of L-Aspartyl-L-phenylalanine, 13th peak), phytosphingosine (14th peak), nicotinic acid (in the form of 6-Hydroxynicotinic acid, 23^rd^ peak), ascorbic acid (in the form of dehydroascorbic acid, 28th peak), and vitamin A (29th peak) in the order of highest to lowest peak intensities. These seven metabolites were previously implicated in the antioxidant and/or anti-inflammatory roles.

## 3. Discussion

In the present study, we demonstrated that the CS water extract ameliorated the hyperproliferation of UVB-induced skin epithelial tissues and wrinkle formation in addition to preserving epidermal collagen content in UVB-irradiated SKH-1 hairless mice. CS extract was also effective in the alteration of Nrf2 and NF-κB target inflammatory genes, which are influenced by oxidative stress, in mouse skin and human skin cells. These anti-UVB effects appeared to be mediated by the antioxidant and anti-inflammatory effects of CS, as shown in mice and in HaCaT cells. 

The UVB-induced skin changes, including skinfold thickness, wrinkle depression volume and epidermal thickness, were ameliorated conspicuously in the CS-treated groups. Reduction in skin photoaging in the CS groups appeared to be due to inhibition of aberrant UVB-induced hyper-proliferation because significant reductions in proliferation markers were detected in the skin of the CS groups. Moreover, prolonged UV exposure in the skin is known to trigger cell proliferation with damaged DNA [1]. However, oral administration of the CS markedly decreased hyperproliferation and DNA damage.

The collagen content in the dermis, which lies below the epidermis, confers resilience and strength to the skin [28]. Continuous UV exposure can lead to the loss of collagen through the reduction in the production of type 1 collagen and increased activities of MMP [28]. In our study, MT and VVG staining showed that collagen fiber was greatly reduced in mice exposed to UVB, but a significant recovery was observed after treatment with CS or its components. The impairment of collagen synthesis by UV irradiation occurs via interference in the TGF-β and Smad2/3 signaling pathway in the skin [3,29]. These aberrations result in a reduction in the phosphorylation of Smad2/3, which consequently decreases the transcription of type 1 procollagen [29]. Remarkable reactivation of TGF-β and Smad2/3 signaling pathway was achieved by the CS in UVB-irradiated mice, suggesting that the synthesis of type 1 procollagen might have been recovered by the CS. Similar results were shown in HaCaT cells, where the CS extract significantly increased the low mRNA levels of procollagen type 1 in UVB-irradiated cells. In addition, expression of MMP-9, which displays proteolytic activities and degrades the extracellular matrix containing collagen and elastin [4], was inhibited by the CS groups. As seen in the CSH group, reduction in TIMP-1, a major inhibitor of MMP-9 [30], might have reflected the condition of lowered MMP-9 to balance the activities of MMPs and TIMPs. Overall the CS prevented the UVB-induced loss of collagen fibers possibly by activating TGF-β and Smad2/3 signaling and inhibiting MMP-9 expression. 

Chronic UVB radiation on skin causes accumulation of ROS, adjacent and tumoral oxidative stress, and oxidative damage [31]. As previously investigated by analytical methods, CS itself or its components exert antioxidant capacities [15,16,17,32]. Antioxidative effects of our CS water extract were in accordance with these studies, showing a dose-dependent increase in radical scavenging capacity. The effects were far lower than that of ascorbic acid, therefore the CS extract may not be considered as a direct antioxidant, but a potential material exerting positive effects on skin protection perhaps with the synergistic effects of various compounds identified in the literature [11,12,13,14]. The radical scavenging activities detected in the CS extract were in agreement with an increase in the murine blood levels of GSH, an antioxidant molecule. GSH acts as a direct scavenger of free radicals [33]. Oxidative stress levels in both blood and skin were lessened by the CS, as shown by the results of the skin 8OHdG, TBARS, and blood comet assays. These antioxidant effects seen in the skin were especially exciting considering that the CS extract was orally administered instead of being applied topically. The UV exposure is known to disturb the antioxidant systems in the body other than the skin and increase oxidative stress markers in the liver and blood [34]. The antioxidative effects observed in the skin tissue and blood might have indicated that the CS reached the skin and blood circulation system and played protective roles in the UVB-induced oxidative stress conditions. Similarly, other studies reported that the oral administration of polyphenol-rich plant extracts prevented UV-induced lipid peroxidation in skin and DNA damage in peripheral blood [35]. The administration of natural food extracts was able to restore the blood GSH concentration in a diabetic animal model [36]. 

Nrf2 is a major regulator of antioxidant responses in cellular level through antioxidant response element (ARE)-mediated transcriptional regulation [37]. Nrf2-deficient mice have shown accelerated oxidative skin damage and photoaging in response to UVB radiation but no difference in carcinogenesis, suggesting that Nrf2 system may play an essential role in relieving UVB-induced oxidative stress in skin [38,39]. Catalase and SOD1 are well known antioxidant enzymes that neutralize excess reactive oxygen species in cells [40]. SOD1 is a direct ROS quencher and its promoter is known to contain Nrf2-ARE binding site [41]. No ARE site has been found in catalase promoter region so direct binding of Nrf2 remains controversial [42], but Nrf2-dependent expression of catalase has been shown in mouse-derived cells [43,44]. We showed that CS extract increased Nrf2 protein in liver tissue and in HaCaT cells, suggesting a positive role of CS in the antioxidant pathway. In accordance with the activation of Nrf2 protein, catalase and SOD1 increased in skin tissue compared with the UVB group. Another Nrf2-regulated antioxidant, glutaredoxin, was reported to alleviate oxidative stress in human retinal pigment epithelial cells [45,46]. We showed that the CS extract significantly improved the mRNA levels compared with that of the UVB group in HaCaT cells. The Nrf2-mediated antioxidant enzyme regulation could have contributed to the photoprotective effect of the CS extract on UVB-irradiated skin. Although we expected CS dose-dependent increases in Nrf-2 protein levels in both animal and cell experiments, we only observed these effects in the cells. The reasons we observed no further increase in protein level of Nrf2 in CSH group could include possible toxic effects of a high dose of CSH itself or effects of negative feedback mechanism after CS intake. According to Heo et al., Nrf2 protein is degraded after antioxidant enzymes [47]. In another study, with increased UVB irradiation, HaCaT cells have been reported to exclude Nrf2 from the nucleus, compared with lower doses of UVB where nuclear translocation was increased [48]. Thus, the Nrf2 protein in the CSH group may have shown no increase due to tight regulation of this protein and possible negative feedback mechanism. 

ROS induced by UVB radiation triggers signaling molecules such as NF-κB, a major regulator of pro-inflammatory genes including iNOS [49]. Inflammatory response caused by UVB-irradiated skin activates the transcription of MMPs, which degrade the dermal collagen and connective tissue in skin [3]. UVB irradiation significantly activated NF-κB signaling, which in turn was blocked only by the higher dose of CS. In contrast, IL-1β and iNOS, which are well-known NF-κB targets [50,51], were successfully downregulated by the CS regardless of the doses in skin and liver tissues. On the other hand COX-2, another NF-κB target [51], was only lowered by the higher dose of CS. In HaCaT cells iNOS was only decreased in the higher dose of CS extract. Collectively, the photoprotective effects of CS might have been involved in the inhibition of UVB-activated NF-κB signaling pathway, leading to the reduction in the expression of proinflammatory genes and MMP-9. 

Metabolites both up-regulated in the skin of the CS group and found in the top 100 peaks in CS extract included proline betaine, L-proline, L-phenylalanine, phytosphingosine, nicotinic acid, ascorbic acid, and vitamin A, in the highest to lowest peak intensity order. Proline and glycine betaine are antioxidants that also protect plants from dehydration [52], salt stress, and cell death [53]. L-phenylalanine exerts lipophilic antioxidant capacity as tested by DPPH and ABTS assays [54] and anti-inflammatory effects on carrageenan-induced edema [55]. Phytosphingosine, an active lipid abundant in both plants and animals, constitutes the stratum corneum (outer layer of skin) and exhibits anti-inflammatory effect and defense against microbes [56]. Phytosphingosine-1-phosphate has been reported to promote epidermal growth factor in human dermal fibroblasts, and promotes anti-aging effects in human skin [57]. Nicotinic acid, known as vitamin B_3_, stimulates keratinocyte differentiation, stabilizes epidermal barrier function, and benefits aging skin by reducing wrinkles and exerting anti-photocarcinogenesis effects [58]. In addition, nicotinic acid has shown anti-inflammatory effects in TNF-α-exposed mouse adipocytes ascorbic acid by suppressing inflammatory chemokines [59]. Ascorbic acid, known as vitamin C, also benefits the skin by promoting collagen formation, scavenging free radicals, and protecting from photoaging and UVB-induced lipid peroxidation [60]. Oral ingestion of vitamin C has been suggested to be more effective on the skin than topical administration [61]. Vitamin A has been effective in alleviating inflammation in skin disorders, broncho-pulmonary dysplasia, and pneumonia [62]. These substances in the CS might have attributed to the UVB protective effects on the skin. 

There are some limitations regarding the measurements of candidate antioxidants in the skin of mice and its molecular relationship with CS’s antioxidant effects. The inclusion of groups for bioactive constituents with the equivalent dose to their content in the CS extract would have provided further support to our study. Previous studies suggested that allantoin [63] and luteolin [64] are present in CS, and may exhibit anti-inflammatory or anti-oxidative effects [65,66,67]. However, allantoin and luteolin in our CS extract were present as the 58th and 3244th most abundant chemical according to our LC-MS/MS analysis and might have not significantly contributed to the CS effects. However, when a 15-fold lower dose of allantoin and a 15-fold higher dose of luteolin were provided to the UVB-irradiated animals, we observed significant improvements in UVB-induced skin damages along with oxidative stress and inflammatory markers (unpublished data). In addition, our CS metabolite analysis suggested other potential antioxidant and/or anti-inflammatory components. Further studies are warranted to identify bioactive constituents for the UVB protective effects of the CS. 

In conclusion, our data demonstrated that the oral administration of the CS extract ameliorated UVB-induced skin photoaging by the prevention of aberrant cell proliferation and DNA damage, and that these effects might be mediated by antioxidant and anti-inflammatory gene pathways. Histological results of skin tissue showed that the CS extract effectively reduced UVB-induced wrinkle formation and cell proliferation, and increased collagen synthesis. Mediators of the antioxidant defense system such as Nrf2, catalase, SOD1, and glutaredoxin were elevated, and inflammation-related genes in the NF-κB signaling pathway, such as IL-1β, COX-2, and iNOS were reduced upon oral ingestion of dietary CS extract. In human cells, similar results were shown in the Nrf2 and NF-κB pathways. As revealed by the LC-MS/MS results, the chemical composition of the CS extract included potential antioxidants, which might have contributed to its anti-photoaging effects in animal tissue and in cells. The results indicate that the CS extract was effective in the prevention of UVB-induced skin damage through different signaling pathways. Further studies on the molecular level of the photoprotective effect of CS water extract on the skin are required.

## 4. Materials and Methods

### 4.1. Materials and Reagents

2,2-Diphenyl-1-picrylhydrazyl (DPPH) was purchased from Alfa Aesar (Haverhill, MA, USA). 2,2-azinobis(3-ethyl-benzothiazoline-6-sulfonic acid) (ABTS), dimethyl sulfoxide (DMSO), and sodium bicarbonate were purchased from Sigma-Aldrich Co. (St. Louis, MO, USA). Potassium persulfate was purchased from Duksan (Ansan-si, Gyeonggi-do, Korea). The assay kits for GSH analysis were purchased from BioVision Research Products (Mountain View, CA, USA). Other chemicals were commercially available and in analytical grade.

### 4.2. Preparation of CS Extract

Water extract of corn silk was prepared and supplied by Kwang Dong Pharmaceutical Co. Ltd. (Seoul, Korea), and stored at 4 °C until use. The corn silk was harvested from Jilin Province in China, sterilized at 125 °C for 30 min, extracted with water at 98 °C–100 °C for 1 h, filtered through microfilter paper, and concentrated under reduced pressure at 50 °C or lower (concentrated brix: 44–48 brix). After final sterilization at 95 °C–98 °C for 20 min, 1 L of corn silk extract was obtained from 4 kg of corn silk. The moisture content of corn silk extract was 64.98%. The extract consisted of 16.21% carbohydrate, 9.78% protein, 0.44% lipid, 5.54% dietary fiber, 0.06% crude fiber, and 8.59%. The nutritional composition of the corn silk extract was determined using the Association of Official Agricultural Chemists (AOAC) method (1996) established by the Korea Health Supplement Institute (Seongnam, Korea) [68]. Finally, lyophilization using a freeze-dryer (FD8512, ilShin BioBase Co. Ltd., Yangju, Korea) for a minimum of 72 h removed water from the extract. In total, 125 g of the freeze-dried corn silk extract (CS) was collected.

### 4.3. Measurement of Antioxidant Effects of CS Extract

To measure the DPPH radical scavenging activity of the CS water extract, 80 μL of DPPH solution (0.4 mM DPPH in ethanol) was vigorously mixed with 20 μL of CS solution (1.25, 2.5, 5, 10 mg/mL in water). For the control, 20 μL water was added to 80 μL DPPH After incubation for 10 min, the absorbance of the solution was measured at 492 nm. The standard content was calculated using the absorbance of L-Ascorbic acid (100 μg/mL) that were treated in the same way as the samples. The ABTS radical scavenging activity was determined by the modified method of Re et al. [69]. In brief, 7 mM ABTS in water was reacted with 2.45 mM potassium persulfate, allowed to stand in dark for 12–16 h to make ABTS•+. This was diluted with methanol (50%, v/v), to absorbance of 0.70 ± 0.02 at 734 nm, 30 °C, and 990 μL of this solution was added to 10 μL CS or water. Absorbance was measured at 734 nm after 1 min incubation. The free radical scavenging ability of DPPH and ABTS was calculated by the following equation: Percentage of inhibition of DPPH or ABTS (%) = (1 – O.D. of sample/O.D. of control) × 100. IC_50_ values of CS extract in relation to ABTS and DPPH free radicals was calculated and compared using L-ascorbic acid (0, 12.5, 25, 50, 100 μg/mL) as a positive control. 

### 4.4. Experimental Animals

Fifty-six 6-week-old female SKH-1 hairless mice were obtained from Orient Bio Inc. (Seongnam, Korea). The animals were housed at 23 ± 2 °C, with a relative humidity of 55 ± 10%, in a 12 h light/dark cycle with free access to food (5L79, Orient Bio Inc., Seongnam, Korea) and water. After a one-week acclimatization, the mice were randomly allocated into six groups: (i) Vehicle (saline)-treated normal group (NOR, *n* = 10), (ii) UVB-irradiated group (UVB, *n* = 9), (iii) UVB-irradiated and 2 g/kg/day CS-treated group (CSL, *n* = 9), and (iv) UVB-irradiated and 4 g/kg/day CS-treated group (CSH, *n* = 10).

The oral dose of each sample was adapted from Guo et al. and Wang et al. at which CS administration did not induce weight loss, histopathological changes, or death [70,71]. The oral dose of the group was set at 4.0 g/kg/day. The oral dose of the low dose group was set at 2.0 g/kg/day. Each sample was dissolved in saline and orally administered at a volume of 0.2 mL each. In the normal and control groups, saline was administered at a dose of 0.2 mL/day. 

To observe the protective effects of the extract on mice before tumorigenesis, we defined 19 weeks as the endpoint of our study in reference to a report that UVB-induced tumor development time in 50% untreated hairless mice was 20 weeks [28]. The animals were monitored daily and weighed weekly. All experimental protocols were approved by the Institutional Animal Care and Use Committee (IACUC) of Yonsei University, Korea (Permit number: 201608-495-02).

Mouse dorsal skin was exposed to UVB three times per week using the Biolink crosslinker BLX-312 (Vilbert Lourmat; Marne-La Vallee, France). Fifteen-centimeter distance was maintained between the light source and mouse. The UVB source was 5 UVB lamps (5 × 8 W [8 J/s]) with a 312 nm peak emission. The minimal erythematous dose was 180 mJ/cm^2^. The UVB radiation was 180 mJ/cm^2^ in weeks 2–11 and increased to 360 mJ/cm^2^ in weeks 12–19 by modified methods of Mantena et al. [72] and Record and Dreosti [73]. No UVB was radiated in the first week.

### 4.5. Assessment of Skin Thickness and Wrinkle Formation in Mice

Hairless mice were anesthetized, and their dorsal skin was photographed at the end of the study (19 weeks). The UV-induced skin fold thickness was measured at a point mid-way between the neck and hips by using an electronic digital micrometer caliper (Marathon Watch Company Ltd., Ontario, Canada). A replica of the mouse dorsal skin was obtained by using silicon rubber (Repliflo, CuDerm Corp., TX, USA). The skin impressions were photographed using an Antera 3D^®^ camera (Miravex, Dublin, Ireland) and analyzed by Antera CS program (Miravex). The volume of the depression, the affected area of depression, maximum depth, and wrinkle length were measured on the surface of the replica (a circle with a 16.8 mm-diameter).

### 4.6. Histological and Immunohistochemical Analysis in Mice

The dorsal skin samples (1 × 1 cm^2^) were fixed in 10% neutralized formalin for at least 24 h and embedded in paraffin. Prior to staining, 4 μm sections were deparaffinized in xylene, and rehydrated through a graded ethanol series (100%–70%). The sections were stained with hematoxylin and eosin (H&E), Masson’s trichrome (MT), and Verhoeffe–Van Gieson (VVG). The immunohistochemical detection of proliferating cell nuclear antigen (PCNA), 8-hydroxy-2′-deoxyguanosine (8-OHdG), and Ki67 was performed in 4 μm thick deparaffinized sections. For antigen retrieval, the sections were incubated with Tris-EDTA buffer solution (pH 9.0) for PCNA or citrate buffer solution (pH 6.0) for 8-OHdG and Ki67 for 15 min in a decloaking chamber (Biocare Medical). The sections were treated with 3% H2O2 for 10 min to inhibit the endogenous peroxidase activity and rinsed in 0.1 M Tris-buffered saline (TBS) solution. Non-specific binding sites were blocked by the incubation of the sections with 5% BSA for 1 h. The sections were incubated with 1:100 diluted anti-8OHdG or anti-Ki67 primary antibody (Abcam, Cambridge, UK) overnight at 4 °C, followed by incubation with the secondary antibody (DAKO Envision+ System-HRP Labelled Polymer: Anti-mouse for PCNA and 8-OHdG, and anti-rabbit for Ki-67) for 1 h. The sections were washed in 0.1 M TBS, incubated with 3,3′-diaminobenzidine tetrahydrochloride peroxidase substrate solution (k-3468, DAKO Corp., CA, USA) for 1–3 min, counterstained with Mayer’s hematoxylin solution, and dehydrated through a graded series of 70%–100% alcohol in xylene. Representative photomicrographs were taken by a light microscope (Eclipse Ti microscope; Nikon, Tokyo, Japan). The epidermal thickness and staining density of collagen were quantified using ImageJ software (National Institutes of Health, MD, USA).

### 4.7. Measurement of Reduced GSH Content in Mouse Plasma

The plasma GSH level was determined by using the Glutathione Fluorometric Assay Kit (BioVision, CA, USA) in accordance with the manufacturer’s instructions. Each sample was added to a 96-well plate. Each well had a total volume of 100 μL containing 20 μL of the plasma from each mouse. Subsequently, 2 μL of GST reagent and 2 μL of monochlorobimane (MCB) was added. After incubation for 1 h at 37 °C, fluorescence was measured by using a GENios fluorescence plate reader at an excitation wavelength of 335 nm and an emission wavelength of 460 nm.

### 4.8. Comet Assay (Alkaline Single-Cell Gel Electrophoresis)

The alkaline comet assay was conducted in accordance with the method of Singh et al., with minor modifications [74]. Leukocytes were isolated from whole blood using Histopaque 1077 (Sigma Aldrich Co.), mixed with 0.7% low melting-point agarose and added to slides. The slides were immersed in cold lysing solution (2.5 M NaCl, 100 mM EDTA, 10 mM Tris, 1% sodium laurylsarcosine, pH 10, 1% Triton X-100, and 10% DMSO) at 4 °C for 1 h. After lysis, the slides were transferred to electrophoresis buffer (300 mM NaOH and 10 mM Na2EDTA, pH 13.0) at 4 °C for 40 min. For electrophoresis, an electric current of 25 V/300 ± 3 mA was applied at 4 °C for 20 min. The slides were washed three times with neutralizing buffer (0.4 M Tris, pH 7.5) at 4 °C and treated with ethanol for 5 min. All steps were conducted in the dark to prevent additional DNA damage. Fifty cells from two replicate slides each were analyzed using a fluorescence microscope (LEICA DM LB, Bensheim, Germany) and image analysis software (Komet 4.0; Kinetic Imaging, UK) to compute the tail intensity (equivalent to the percentage of DNA in the tail), tail length, and tail moment (tail length × tail intensity).

### 4.9. Determination of Lipid Peroxidation in Skin Tissue

Mouse skin tissue was homogenized in 1.15% KCl solution (30 mg/mL). After centrifugation (1,500 g, 15 min, 4 °C), 100 μL supernatant was reacted with 400 μL of TBARS reagent (0.8% TBA) and incubated at 95 °C for 1 h. To terminate the reaction, 250 μL of distilled water and 1.25 mL of n-butanol/pyridine (15:1, v/v) were added, and the solution was centrifuged again (1,500 g, 15 min). The absorbance of the supernatant was measured at 540 nm and the concentration of lipid peroxides was calculated based on the malondialdehyde (MDA) standard curve and normalized to the protein content of each sample by the bicinchoninic acid assay (BCA; Pierce, Rockford, USA).

### 4.10. Cell Culture

HaCaT cells (Item no. 300493, Cell Lines Service, Eppelheim, Germany) were cultured in Dulbecco’s modified Eagle medium nutrient mixture F-12 (Ham) 1:1 (D-MEM/F-12, GibcoBRL, Braunschweig, Germany) powder medium supplemented with 10% fetal calf serum (FCS) (GibcoBRL, Grand Island, NY, USA), 1% antibiotic/antimycotic solution (Corning, NY, USA) and 1.2 g/L sodium bicarbonate (NaHCO3; Sigma-Aldrich, cat. No. S6014). Cells were incubated in 5% CO_2_ at 37 °C.

Cells were irradiated with UVB in Chambres Noires darkrooms (Vilber, France) under a UVB light lamp (VL-6 MC; Vilber, France) emitting 315 nm. The UVB emission was calculated as 30 mJ/cm^2^ = 580 µW/cm^2^ × 52 s. 

### 4.11. Cell Viability (MTT) Assay

Cytotoxicity of CS and UVB on HaCaT cells was measured by colorimetric MTT assay. Cells were seeded in 96-well plates to make 2 × 10^4^ cells/well. After 24 h incubation in complete medium, water (control) or 0.1~15 μg/mL CS extract was added to each well. After 24 h incubation, cells were washed and either replaced with fresh complete medium or UVB-irradiated at 30, 45, and 70 mJ/cm^2^ (52, 77, 120 s) in 200 μL PBS and then replaced with fresh complete medium with the same concentration of CS extract or water (control). After 5 h incubation, 1 mg/mL MTT solution was added to each well and allowed to react for 4 h. The supernatant was removed and 100 μL of isopropyl alcohol (Merck, Darmstadt, Germany) was added to dissolve the generated formazan crystals. The absorbance was measured at 570 nm using a microplate reader (Infinite^®^ 200 PRO, Tecan, Switzerland). 

### 4.12. Western Blot Analysis

The UVB-irradiated total dorsal skin was homogenized (Polytron System PT 1200 E, Luzernerstrasse, Switzerland) in RIPA lysis buffer with freshly added protease and phosphatase inhibitor cocktails. After centrifugation (4 °C, 12,500 g, 20 min), the supernatant protein concentration was determined by using the Bradford reagent (BioRad, CA, USA). Equal amounts of total protein were separated by SDS-PAGE and transferred to PVDF membranes (Millipore Corporation). Next, the membranes were blocked by incubation in 5% fat-free dry milk in 1× phosphate-buffered saline (PBS) for 1 h at room temperature. The membranes were then incubated overnight 4 °C with 1:1000 dilutions of the primary antibodies specific for α-tubulin, IL-1β, PCNA, MMP9, TIMP-1, Smad2, p-Smad2 (Santa Cruz Biotechnology, Dallas, TX, USA), iNOS, COX-2 (BD Biosciences, San Jose, CA, USA), TGF-β (R&D Systems Inc., Minneapolis, MN, USA), 8OHdG (Abcam, Cambridge, UK), Ki67 (Millipore Corporation, Billerica, MA, USA), and Nrf2 (Invitrogen, MA, USA). The membranes were rinsed with PBST and incubated with peroxidase-conjugated 1:5000 dilutions of the anti-mouse and anti-rabbit secondary antibodies (Millipore Corporation, Billerica, MA, USA). The antibody signals were visualized by a chemiluminescence detection system (GE Healthcare Life Sciences, Buckinghamshire, England) and photographed by an AE-9300 Ez-Capture system (ATTO, Tokyo, Japan). Band density was quantified using ImageJ software.

HaCaT cells were seeded in a 60 mm culture dish to make 8 x 10^5^ cells/well and incubated in complete media for 24 h. Then, water (NOR, UVB), 0.5 μg/mL CS extract or 5 μg/mL CS extract was treated and incubated for 24 h. Then, cells were washed with PBS and replaced with fresh complete medium (NOR) or UVB-irradiated at 315 nm for 52 s at 30 mJ/cm^2^ in 2 mL PBS, and then replaced with fresh complete medium with the same concentration of CS extract or water (control). After 5 h incubation, protein was extracted from the cells in RIPA lysis buffer for Western blot analysis. 

### 4.13. Reverse Transcriptase (RT) and Quantitative Polymerase Chain Reaction (qPCR)

Skin tissue total RNA was extracted by homogenization in TRIzol reagent (MRC, Cincinnati, OH, USA. Cell total RNA was extracted by scrapping in TRIzol reagent (MRC, Cincinnati, OH, USA). The purity of the total RNA was measured with a spectrophotometer and reverse transcribed using ImProm II Reverse Transcriptase kit (Promega, Madison, Wis., USA). cDNA was synthesized according to the manufacturer’s protocol. qPCR was performed in a Mic real-time PCR system (BMS, biomolecular systems, Australia) using 5x HOT FIREPOL^®^ EvaGreen^®^ qPCR Supermix (Solis biodyne, Tartu, Estonia) in a volume of 18 uL depending on the manufacturer’s cycling conditions. Relative gene expression was measured using the comparative 2-(ΔΔCq) method. Expression of housekeeping GAPDH mRNA was used for qPCR data standardization. The primers used are shown in Table 2. 

### 4.14. Sample Preparation for Liquid Chromatography-Tandem Mass Spectrometer (LC-MS/MS) Analysis

Non-targeted LC-MS/MS was used to analyze the components present in the CS extract. The CS extract was diluted 10,000 times with deionized water and filtered 0.45 μm. CS extract samples were stored at −20 °C until LC-MS/MS analysis.

### 4.15. LC-MS/MS Analysis and Identification of Metabolites

Ultimate 3000 UHPLC and Q-Extractive Orbitrap Plus was equipped with an ACQUITY C18 column (10 cm × 2.1 mm, particle size 1.7 μm, Waters, USA). The injection volume for each sample was 5 μL. The column was eluted using the following binary gradient solutions: A (deionized water with 0.1% formic acid) and B (methanol, UPLC graded) with a flow rate of 0.4 mL/min, A: B = 100: 0 at 1 min, 0: 100 at 16 min, 0: 100 until 20 min, and 100: 0 at 22 min. The full scan/dd-MS2 system parameters were: FTMS, ESI-positive mode with mass resolution of 70,000, full scan range: 80~1000 m/z, dd-MS2 (Top 10) resolution of 17,500 with collision energy 30, flow rate of nitrogen sheath gas and auxiliary gas: 40 (arbitrary units) and 10 (arbitrary units), spray voltage: 3.5 kV, capillary temperature: 320 °C, S-lensRF level: 50, auxiliary gas heater temperature: 300 °C. Metabolomics analysis for mouse skin was carried out as outlined in the supplementary methods.

The original data from the UPLC-Orbitrap-MS2 analysis were extracted from the XCMS online platform (http://xcmsonline.scripps.edu/) for data alignment and peak detection. The parameters of the XCMS online were 10-sec bandwidth, 15 ppm tolerance for database research, and the rest were UPLC-orbitrap default values. The MS2 data for peak identification was extracted from the LC-raw file using Xcalibur 2.2 (Thermo Fisher Scientific, San Jose, CA, USA). The LC-MS/MS search function of the online database (MycompoundID (www.mycompoundid.org) and HMDB (www.hmdb.ca)) was used to perform metabolite identification using retention time, exact mass, and MS/MS peak intensities. 

### 4.16. Statistical Analysis

The data are expressed as mean ± standard error (SE) or standard deviation (SD). The results were analyzed by one-way analysis of variance (ANOVA) and one tail t-test using SPSS software (SPSS, Version 23.0 IBM Inc., USA). The criterion for statistical significance was *p* < 0.05.

## Figures and Tables

**Table 1:** IC_50_ values of DPPH and ABTS radical scavenging activities of corn silk (CS) extract and ascorbic acid.

Sample	IC_50_ (mg/mL)	
	DPPH	ABTS
Corn silk extract	3.60 ± 0.1	11.61 ± 0.2
L-ascorbic acid	0.08 ± 0.0	0.38 ± 0.1

Values are represented as mean ± SD of three replicates. Lower values represent higher radical scavenging activity of the corresponding sample.

**Table 2:** Primers used for quantitative polymerase chain reaction (qPCR).

Gene	Forward Sequence	Reverse Sequence	Product Size
m.Catalase	AACGCTGGATGGATTCTCCC	GCCCTAACCTTTCATTTCCCTTCAG	133
m.Procollagen type 1	CCCTAGCCTTTTCTCCGCC	TGGCAACTCCAAGTCCATCAT	238
m.Nrf2	GTGAGACGTGGAAACCCGAG	GCCATAGGACATCTGGGAAGC	347
m.TXN	GAGCAAGGAAGCTTTTCAGGAG	GTCCCGTTTTGGATCCGAGT	252
m.SOD1	ATGGCGACGAAGGCCGTGTG	GACCACCAGTGTGCGGCCAA	360
m.GAPDH	AAGGTCGGTGTGAACGGATTT	CAGAAGGGGCGGAGATGATG	364
h.Glutaredoxin	CATCGGCATGGCTCAAGAG	AATCTGCTTTAGCCGCGTCA	313
h.Procollagen type 1	AGGACAAGAGGCATGTCTGGTT	TTGCAGTGTAGGTGATGTTCTG	156
h.GAPDH	AAGGTCGGTGTGAACGGATTT	CAGAAGGGGCGGAGATGATG	364

## Supplementary Materials

The following are available online at https://www.mdpi.com/1420-3049/24/14/2587/s1, Table S1: Compounds identified in corn silk (CS) extract and mouse skin using LC-MS/MS; Figure S1: Heatmaps of UVB-irradiated and CS extract-administered mouse skin metabolites.
