# Supplementary material for: Potential Photoprotective Effect of Dietary Corn Silk Extract on Ultraviolet B-Induced Skin Damage
Source: Molecules. 2019 Jul 16;24(14):2587. doi: 10.3390/molecules24142587 (PMC6680388; doi:10.3390/molecules24142587)
Supplement: Supplementary file 1 [file molecules-24-02587-s001.zip › Supplementary Methods-final.v2.pdf]

Supplementary Methods

Mouse skin sample preparation for liquid chromatography-tandem mass spectrometer (LC-MS/MS) analysis

For skin metabolites extraction, 60 mg of skin tissues were minced and homogenized for 5 min in 600 μl 100 % methanol on ice using a disposable homogenizer (BioMasher II, Nippi, Inc., Tokyo, Japan). Next, 10 μM 4-(2-hydroxyethyl)-1-piperazineethanesulfonic acid (HEPES) and piperazine-N,N′-bis(2-ethanesulfonic acid (PIPES) in 100% methanol were added to the samples as internal standards. HEPES and PIPES were used as internal standards to correct for the variation of sample preparation and normalization. After quick vortexing, samples were centrifuges at 12,000 rpm for 10 minutes at 4 °C. Supernatant was lyophilized overnight. Each sample was resuspended in 600 μl 100 % methanol and filtered with 0.2 μm polytetrafluoethylene (PTFE). Skin metabolites extraction samples were used for LC-MS/MS analysis.

Statistical Analysis for mouse skin metabolomics analysis

For all metabolites, univariate Mann-Whitney *U*-test and multivariate orthogonal partial least squares discriminant analysis (OPLS-DA) were performed (SIMCA 14.1, Umetrics Inc., Umea, Sweden). Logarithmic transformation and pareto scaling were carried out before multivariate analysis. The goodness of fit was indicated by R^2X and R^2Y, and the predictive ability was assessed by Q^2Y parameters. The meaningful metabolites were listed according to the following parameters, including a univariate p-value of < 0.05 and multivariate variable importance in the projection (VIP) value of >1.0. For drawing the heatmaps, logarithmic transformation and pareto scaling were carried out using Metaboanalyst 4.0, an open-access online tool (www.metaboanalyst.ca).
