# Supplementary material for: Potential Photoprotective Effect of Dietary Corn Silk Extract on Ultraviolet B-Induced Skin Damage
Source: Molecules. 2019 Jul 16;24(14):2587. doi: 10.3390/molecules24142587 (PMC6680388; doi:10.3390/molecules24142587)
Supplement: Supplementary file 1 [file molecules-24-02587-s001.zip › Supplementary Tables and Figures-final.pdf]

Table S1. Compounds identified in corn silk (CS) extract and mouse skin using LC-MS/MS

			CS extract	CS mouse skin (CSL)	CS mouse skin (CSH)				CS extract	CS mouse skin (CSL)	CS mouse skin (CSH)
No.	ID	Metabolites	Intensities	Intensities	Intensities	No.	ID	Metabolites	Intensities	Intensities	Intensities
1	HMDB00738	**Indole** [1,2]	330.24×10^7			51	HMDB00126	Glycerol 3-phosphate	12.16×10^7	0.75×10^7	0.73×10^7
2	HMDB00375	**3-(3-Hydroxyphenyl)propanoic acid** [3]	211.97×10^7			52	HMDB04063	Metanephrine	12.12×10^7	1.49×10^7	2.14×10^7
3	HMDB00894	Vinylacetylglycine	153.76×10^7	11.42×10^7	9.58×10^7	53	HMDB00482	Caprylic acid	11.56×10^7		
4	HMDB01412	7,8-Dihydropteroic acid	142.74×10^7	0.15×10^7	0.16×10^7	54	HMDB06488	N-Acetyl-L-glutamate 5-semialdehyde	10.42×10^7		
5	HMDB12148	2-Hexaprenyl-6-methoxy-1,4-benzoquinol	118.31×10^7	0.96×10^7	0.98×10^7	55	HMDB12138	1-Naphthol	10.06×10^7		
6	HMDB00452	L-Alpha-aminobutyric acid	115.6×10^7			56	HMDB04821	Bisnorbiotin	10×10^7	2.65×10^7	1.65×10^7
7	HMDB04827	**Proline betaine** [4,5]	97.09×10^7	0.06×10^7	0.05×10^7	57	HMDB01552	2-Keto-glutaramic acid	9.64×10^7		
8	HMDB00167	**L-Threonine** [6,7]	91.34×10^7	0.09×10^7	0.16×10^7	58	HMDB00462	Allantoin	9.57×10^7	1.27×10^7	1.74×10^7
9	HMDB00687	**L-Leucine** [8,9]	82.09×10^7			59	HMDB00510	Aminoadipic acid	9.51×10^7		
10	HMDB00930	**trans-Cinnamic acid** [10],[11,12]	70.9×10^7			60	HMDB09449	PE(20:5/14:1)	9.44×10^7		
11	HMDB00162	**L-proline** [13],[14]	66.31×10^7	6.6×10^7	4.74×10^7	61	HMDB33433	Homostachydrine	9.3×10^7		
12	HMDB00267	**Pyroglutamic acid** [15],[16]	65.16×10^7	0.81×10^7	0.38×10^7	62	HMDB07295	DG(18:3/22:6)	9.16×10^7		
13	HMDB00159	**L-Phenylalanine** [17],[18]	49.79×10^7	0.38×10^7	0.3×10^7	63	HMDB13164	2-Hydroxylauroylcarnitine	8.18×10^7	0.51×10^7	0.87×10^7
14	HMDB04610	**Phytosphingosine** [19],[20]	48.96×10^7	0.18×10^7	0.14×10^7	64	HMDB0012204	Cis-zeatin	8.93×10^7	0.1×10^7	0.3×10^7
15	HMDB00174	L-Fucose	45.1×10^7			65	HMDB00500	4-Hydroxybenzoic acid	8.11×10^7	0.95×10^7	1.11×10^7
16	HMDB00962	**Lipoamide** [21]	37.85×10^7	0.88×10^7	0.75×10^7	66	HMDB00472	5-Hydroxy-L-tryptophan	8.09×10^7	0.38×10^7	1.39×10^7
17	HMDB00262	Thymine	37.34×10^7			67	HMDB00562	Creatinine	7.99×10^7	9.35×10^7	15.32×10^7
18	HMDB00191	L-Aspartic acid	36.27×10^7	2.46×10^7	3.31×10^7	68	HMDB00050	Adenosine	7.73×10^7	0.32×10^7	0.37×10^7
19	HMDB01438	**25-Hydroxyvitamin D2** [22]	35.19×10^7	0.19×10^7	0.21×10^7	69	HMDB00567	Cinnamic acid	7.5×10^7	0.98×10^7	1.17×10^7
20	HMDB01713	m-Coumaric acid	34.56×10^7			70	HMDB12096	SM(d18:1/12:0)	7.39×10^7	0.52×10^7	0.8×10^7
21	HMDB00707	4-Hydroxyphenylpyruvic acid	34.07×10^7			71	HMDB04058	5,6-Dihydroxyindole	7.15×10^7	0.53×10^7	0.35×10^7
22	HMDB00703	**Mandelic acid** [10],[23]	31.96×10^7			72	HMDB00444	3-Furoic acid	7.11×10^7		
23	HMDB01488	**Nicotinic acid** [24],[25],[26]	31.25×10^7	15.95×10^7	11.85×10^7	73	HMDB06115	Benzaldehyde	6.98×10^7	6.61×10^7	7.08×10^7
24	HMDB00832	Capryloylglycine	31.1×10^7	38.44×10^7	38.79×10^7	74	HMDB02331	Imidazoleacetic acid riboside	6.61×10^7		
25	HMDB00070	Pipecolic acid	28.32×10^7			75	HMDB01169	4-Aminophenol	6.26×10^7		
26	HMDB01410	2-Amino-4-oxo-6-(1',2'-dioxoprolyl)-7,8-dihydroxypteridine	26.21×10^7			76	HMDB00232	Quinolinic acid	6.14×10^7		
27	HMDB00157	**Hypoxanthine** [27],Abcam(143643)]	25.74×10^7	42.82×10^7	49.7×10^7	77	HMDB07005	CPA(18:1)	6.05×10^7	0.17×10^7	0.37×10^7

28	HMDB00044	**Ascorbic acid** [28],[29,30]	25.63×10^{7}	0.12×10^{7}	0.15×10^{7}	78	HMDB02287	Homocysteine thiolactone	5.99×10^{7}	0.1×10^{7}	0.1×10^{7}
29	HMDB00305	**Vitamin A** [31],[32]	24.04×10^{7}	0.2×10^{7}	0.45×10^{7}	79	HMDB01334	Tridemorph	5.66×10^{7}	2.75×10^{7}	3.27×10^{7}
30	HMDB00020	**p-Hydroxyphenylacetic acid** [33],[34]	23.24×10^{7}	0.5×10^{7}	0.52×10^{7}	80	HMDB07918	PC(14:1/20:5)	5.63×10^{7}	0.32×10^{7}	0.25×10^{7}
31	HMDB12234	Histidinal	23.03×10^{7}	1.45×10^{7}	1.43×10^{7}	81	HMDB01488	Nicotinic acid	5.63×10^{7}	4.88×10^{7}	4.3×10^{7}
32	HMDB00034	Adenine	22.59×10^{7}	1.07×10^{7}	1×10^{7}	82	HMDB01527	3-Methylthiopropionic acid	5.41×10^{7}	3.15×10^{7}	2.11×10^{7}
33	HMDB03011	O-Acetylserine	21.18×10^{7}			83	HMDB03441	Cinnamaldehyde	5.07×10^{7}		
34	HMDB34610	Zucchini factor b	20.36×10^{7}			84	HMDB12228	Ethylphosphate	4.99×10^{7}	0.15×10^{7}	0.11×10^{7}
35	HMDB02511	3,4,5-Trimethoxycinnamic acid	20.05×10^{7}	3.87×10^{7}	3.78×10^{7}	85	HMDB02149	3 Hydroxycoumarin	4.79×10^{7}	7.54×10^{7}	7.97×10^{7}
36	HMDB01476	3-Hydroxyanthranilic acid	17.21×10^{7}	0.22×10^{7}	0.26×10^{7}	86	HMDB03573	Scopolamine	4.49×10^{7}	1.32×10^{7}	0.49×10^{7}
37	HMDB04072	4-Hydroxystyrene	17.04×10^{7}			87	HMDB00651	Decanoylcarnitine	4.45×10^{7}	1.37×10^{7}	1.24×10^{7}
38	HMDB02107	Phthalic acid	16.97×10^{7}			88	HMDB01232	4-Nitrophenol	4.36×10^{7}	1.82×10^{7}	2.59×10^{7}
39	HMDB02268	Beta-Cryptoxanthin	16.04×10^{7}	0.08×10^{7}	0.17×10^{7}	89	HMDB00707	4-Hydroxyphenylpyruvic acid	4.36×10^{7}		
40	HMDB00043	Betaine	15.64×10^{7}	2.03×10^{7}	2.14×10^{7}	90	HMDB60681	4-hydroxy-2,6-dimethylaniline	4.32×10^{7}		
41	HMDB10700	CerP(d18:1/16:0)	15.57×10^{7}			91	HMDB02080	Petroselinic acid	4.29×10^{7}	103.93×10^{7}	60.9×10^{7}
42	C05908 (KEGG)	Delphinidin	14.28×10^{7}			92	HMDB02285	2-Indolecarboxylic acid	4.22×10^{7}		
43	HMDB00158	L-Tyrosine	14.26×10^{7}	0.14×10^{7}	0.12×10^{7}	93	HMDB12182	8-Hydroxypurine	4.1×10^{7}		
44	HMDB00148	L-Glutamic acid	14.14×10^{7}			94	HMDB12273	Palmitic amide	4.1×10^{7}	0.39×10^{7}	0.46×10^{7}
45	HMDB01868	5-Methoxysalicylic acid	13.98×10^{7}			95	HMDB00130	Homogentisic acid	4.09×10^{7}	0.58×10^{7}	0.57×10^{7}
46	HMDB00014	Deoxycytidine	13.73×10^{7}	6.23×10^{7}	5.9×10^{7}	96	HMDB02144	1,3-Dimethyluracil	4.08×10^{7}	0.65×10^{7}	0.39×10^{7}
47	HMDB00227	Mevalonic acid	13.66×10^{7}	18.76×10^{7}	19.82×10^{7}	97	HMDB01127	6-Phosphonoglucono-D-lactone	3.96×10^{7}		
48	HMDB01388	Alpha-Linolenic acid	13.62×10^{7}			98	HMDB00734	Indoleacrylic acid	3.96×10^{7}	0.96×10^{7}	1.08×10^{7}
49	HMDB11714	Vanilpyruvic acid	12.71×10^{7}	0.06×10^{7}	0.1×10^{7}	99	HMDB02287	Homocysteine thiolactone	3.85×10^{7}	0.1×10^{7}	0.1×10^{7}
50	HMDB06833	2-Acetolactate	12.34×10^{7}			100	HMDB12199	Chorismate	3.82×10^{7}	0.16×10^{7}	0.12×10^{7}

Metabolite identification was identified based on accurate mass data, retention time, experimental MS/MS spectra, and library MS/MS spectra in HMDB, MyCompoundID, and KEGG. Compounds detected with the highest to the 100th maximum intensity were listed in intensity-based order. Compounds marked **bold** are metabolites exhibiting antioxidative and anti-inflammatory and photo-aging effects in corn silk (CS) extract among the top 30 peaks.

1. Biradar, J.; Sasidhar, B.; Parveen, R. Synthesis, antioxidant and DNA cleavage activities of novel indole derivatives. *European Journal of Medicinal Chemistry* **2010**, *45*, 4074-4078.
2. Fernandes, E.; Costa, D.; Toste, S.A.; Lima, J.L.; Reis, S. In vitro scavenging activity for reactive oxygen and nitrogen species by nonsteroidal anti-inflammatory indole, pyrrole, and oxazole derivative drugs. *Free Radical Biology and Medicine* **2004**, *37*, 1895-1905.
3. Dueñas, M.; Surco-Laos, F.; González-Manzano, S.; González-Paramás, A.M.; Santos-Buelga, C. Antioxidant properties of major metabolites of quercetin. *European Food Research and Technology* **2011**, *232*, 103-111.

4. Banu, M.N.A.; Hoque, M.A.; Watanabe-Sugimoto, M.; Matsuoka, K.; Nakamura, Y.; Shimoishi, Y.; Murata, Y. Proline and glycinebetaine induce antioxidant defense gene expression and suppress cell death in cultured tobacco cells under salt stress. *Journal of Plant Physiology* **2009**, *166*, 146-156, doi:https://doi.org/10.1016/j.jplph.2008.03.002.
5. Zhao, G.; He, F.; Wu, C.; Li, P.; Li, N.; Deng, J.; Zhu, G.; Ren, W.; Peng, Y. Betaine in inflammation: mechanistic aspects and applications. *Frontiers in immunology* **2018**, *9*.
6. Habte-Tsion, H.-M.; Ren, M.; Liu, B.; Ge, X.; Xie, J.; Chen, R. Threonine modulates immune response, antioxidant status and gene expressions of antioxidant enzymes and antioxidant-immune-cytokine-related signaling molecules in juvenile blunt snout bream (Megalobrama amblycephala). *Fish & shellfish immunology* **2016**, *51*, 189-199.
7. Gaifem, J.; Gonçalves, L.G.; Dinis-Oliveira, R.J.; Cunha, C.; Carvalho, A.; Torrado, E.; Rodrigues, F.; Saraiva, M.; Castro, A.G.; Silvestre, R. L-Threonine supplementation during colitis onset delays disease recovery. *Frontiers in physiology* **2018**, *9*.
8. Cojocaru, E.; Filip, N.; Ungureanu, C.; Filip, C.; Danciu, M. Effects of valine and leucine on some antioxidant enzymes in hypercholesterolemic rats. *Health* **2014**, *6*, 2313.
9. Humberto Nicastro, M.C., Gustavo Barquilha and Leila Soares Ferreira. Leucine Supplementation: A Possible Anti-Inflammatory Strategy Evidences from a

Pilot Study. *Nutrition and metabolism* **2017**, *1*, 114.
10. Li, W.; Zhao, X.; Sun, X.; Zu, Y.; Liu, Y.; Ge, Y. Evaluation of antioxidant ability in vitro and bioavailability of trans-cinnamic acid nanoparticle by liquid antisolvent precipitate. *Journal of Nanomaterials* **2016**, *2016*, 84.
11. Godoy, M.; Rotelli, A.; Pelzer, L.; Tonn, C. Antiinflammatory activity of cinnamic acid esters. *Molecules* **2000**, *5*, 547-548.
12. Hseu, Y.-C.; Korivi, M.; Lin, F.-Y.; Li, M.-L.; Lin, R.-W.; Wu, J.-J.; Yang, H.-L. Trans-cinnamic acid attenuates UVA-induced photoaging through inhibition of AP-1 activation and induction of Nrf2-mediated antioxidant genes in human skin fibroblasts. *Journal of dermatological science* **2018**, *90*, 123-134.
13. Soshinkova, T.; Radyukina, N.; Korolkova, D.; Nosov, A. Proline and functioning of the antioxidant system in Thellungiella salsuginea plants and cultured cells subjected to oxidative stress. *Russian journal of plant physiology* **2013**, *60*, 41-54.
14. Andrade, V.S.; Rojas, D.B.; de Andrade, R.B.; Kim, T.D.H.; Vizuete, A.F.; Zanatta, Â.; Wajner, M.; Gonçalves, C.-A.S.; Wannmacher, C.M.D. A possible anti-inflammatory effect of proline in the brain cortex and cerebellum of rats. *Molecular neurobiology* **2018**, *55*, 4068-4077.
15. Montenegro, L.; Panico, A.; Santagati, L.; Siciliano, E.; Intagliata, S.; Modica, M. Solid lipid nanoparticles loading idebenone ester with pyroglutamic acid: In vitro antioxidant activity and in vivo topical efficacy. *Nanomaterials* **2019**, *9*, 43.
16. Gong, Q.; Li, L.; Wu, X.; Ma, H. Pyroglutamate aminopeptidase 1 may be an indicator of cellular inflammatory response as revealed using a sensitive long-wavelength fluorescent probe. *Chemical science* **2016**, *7*, 4694-4697.
17. Nausheen Joondan, S.B.J.L., Prakashanand Caumul, Prashant Suresh Kharkar. Antioxidant, Antidiabetic and Anticancer Activities of L-Phenylalanine and L-Tyrosine Ester Surfactants: In vitro and In Silico Studies of their Interactions with Macromolecules as Plausible Mode of Action for their Biological Properties. *Current Bioactive Compounds*.
18. Saxena, R.; Pendse, V.; Khanna, N. Anti-inflammatory and analgesic properties of four amino-acids. *Indian journal of physiology and pharmacology* **1984**, *28*, 299-305.
19. Pavicic, T.; Wollenweber, U.; Farwick, M.; Korting, H. Anti-microbial and-inflammatory activity and efficacy of phytosphingosine: an in vitro and in vivo study addressing acne vulgaris. *International journal of cosmetic science* **2007**, *29*, 181-190.
20. Kwon, S.B.; An, S.; Kim, M.J.; Kim, K.R.; Choi, Y.M.; Ahn, K.J.; An, I.-S.; Cha, H.J. Phytosphingosine-1-phosphate and epidermal growth factor synergistically restore extracellular matrix in human dermal fibroblasts in vitro and in vivo. *International journal of molecular medicine* **2017**, *39*, 741-748.
21. Nordman, T.; Xia, L.; Björkhem-Bergman, L.; Damdimopoulos, A.; Nalvarte, I.; Arnér, E.S.; Spyrou, G.; Eriksson, L.C.; Björnstedt, M.; Olsson, J.M. Regeneration of the antioxidant ubiquinol by lipoamide dehydrogenase, thioredoxin reductase and glutathione reductase. *Biofactors* **2003**, *18*, 45-50.
22. Mousa, A.; Misso, M.; Teede, H.; Scragg, R.; de Courten, B. Effect of vitamin D supplementation on inflammation: protocol for a systematic review. *BMJ open* **2016**, *6*, e010804.
23. Benefits of Mandelic Acid. *School of Botanical & Medical Aesthetics* **2015**.
24. Kamat, J.; Devasagayam, T. Nicotinamide (vitamin B3) as an effective antioxidant against oxidative damage in rat brain mitochondria. *Redox Report* **1999**, *4*, 179-184.
25. Digby, J.E.; McNeill, E.; Dyar, O.J.; Lam, V.; Greaves, D.R.; Choudhury, R.P. Anti-inflammatory effects of nicotinic acid in adipocytes demonstrated by suppression of fractalkine, RANTES, and MCP-1 and upregulation of adiponectin. *Atherosclerosis* **2010**, *209*, 89-95.

26. Gehring, W. Nicotinic acid/niacinamide and the skin. *Journal of cosmetic dermatology* **2004**, *3*, 88-93.
27. Mesquita Casagrande, A.C.; Wamser, M.N.; de Lima, D.D.; Pereira da Cruz, J.G.; Wyse, A.T.; Dal Magro, D.D. In vitro stimulation of oxidative stress by hypoxanthine in blood of rats: prevention by vitamins e plus C and allopurinol. *Nucleosides, Nucleotides and Nucleic Acids* **2013**, *32*, 42-57.
28. Barrita, J.L.S.; Sánchez, M.d.S.S. Antioxidant role of ascorbic acid and his protective effects on chronic diseases. In *Oxidative Stress and Chronic Degenerative Diseases-A Role for Antioxidants*, IntechOpen: 2013.
29. Ellulu, M.S.; Rahmat, A.; Patimah, I.; Khaza'ai, H.; Abed, Y. Effect of vitamin C on inflammation and metabolic markers in hypertensive and/or diabetic obese adults: a randomized controlled trial. *Drug design, development and therapy* **2015**, *9*, 3405.
30. Pullar, J.; Carr, A.; Vissers, M. The roles of vitamin C in skin health. *Nutrients* **2017**, *9*, 866.
31. Dao, D.Q.; Ngo, T.C.; Thong, N.M.; Nam, P.C. Is Vitamin A an Antioxidant or a Pro-oxidant? *The Journal of Physical Chemistry B* **2017**, *121*, 9348-9357, doi:10.1021/acs.jpcb.7b07065.
32. Reifen, R. Vitamin A as an anti-inflammatory agent. *Proceedings of the Nutrition Society* **2002**, *61*, 397-400, doi:10.1079/PNS2002172.
33. Jung, Y.-S.; Kang, T.-S.; Yoon, J.-H.; Joe, B.-Y.; Lim, H.-J.; Seong, C.-M.; Park, W.-K.; Kong, J.-Y.; Cho, J.; Park, N.-S. Synthesis and evaluation of 4-hydroxyphenylacetic acid amides and 4-hydroxycinnamamides as antioxidants. *Bioorganic & medicinal chemistry letters* **2002**, *12*, 2599-2602.
34. Liu, Z.; Xi, R.; Zhang, Z.; Li, W.; Liu, Y.; Jin, F.; Wang, X. 4-hydroxyphenylacetic acid attenuated inflammation and edema via suppressing HIF-1α in seawater aspiration-induced lung injury in rats. *International journal of molecular sciences* **2014**, *15*, 12861-12884.

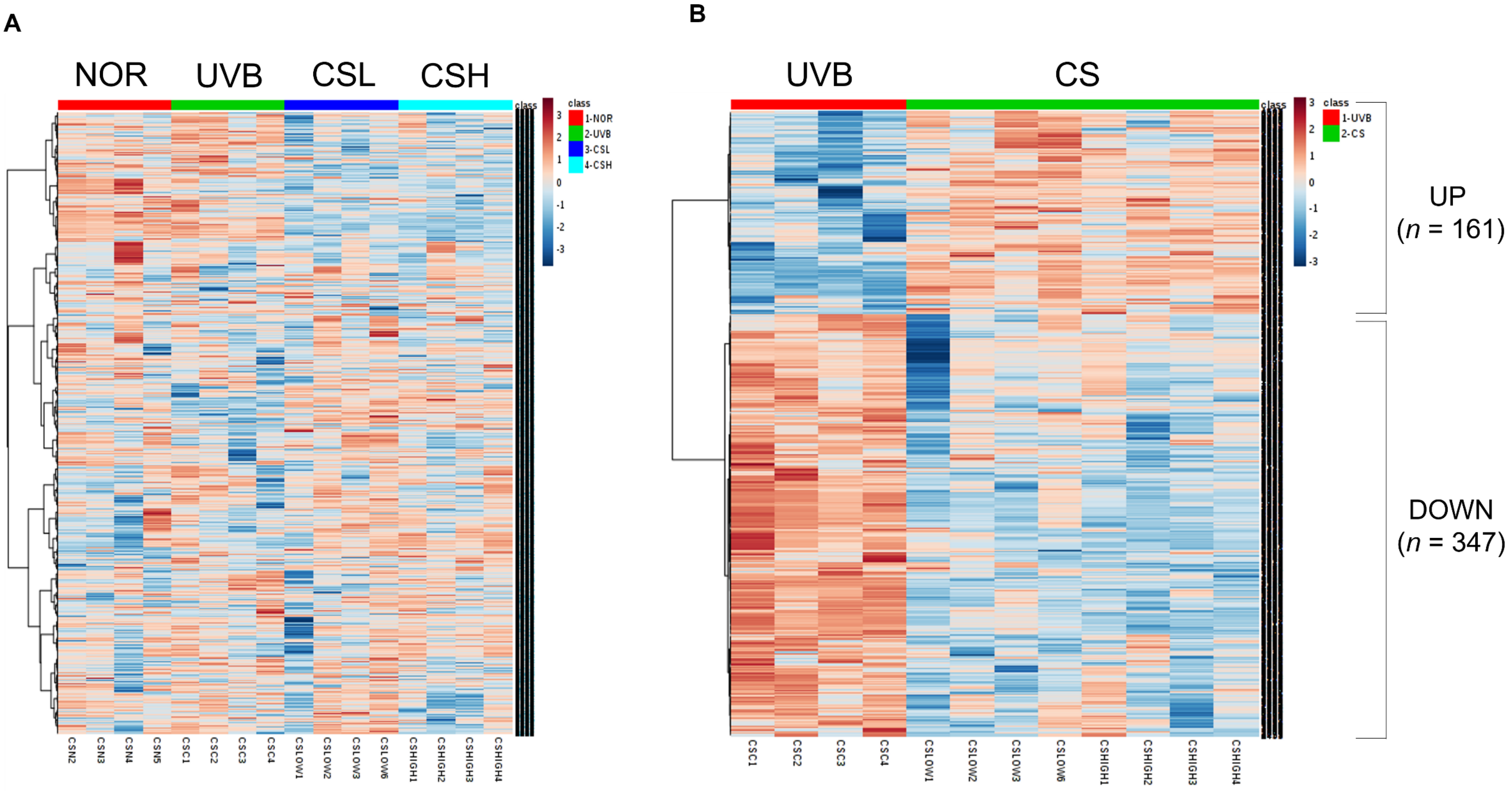

Figure S1. Heatmaps of UVB-irradiated and CS extract-administered mouse skin metabolites.

A total of 7886 peaks were identified by an LC-MS/MS based metabolomics analysis, as shown by a heatmap illustrating the change in metabolite peak intensities in normal mice (NOR), UVB-irradiated mice (UVB), UVB-irradiated and 2.0g/kg/day CS-treated mice (CSL), and UVB-irradiated and 4.0g/kg/day CS-treated mice (CSH) (A). The UVB group and both CSL, CSH groups combined into CS group were compared in heatmap form to show upregulated (UP) and downregulated (DOWN) metabolites in mics skin (B). A total of 508 metabolites were compared after data filtering by VIP > 1.0 from OPLS-DA and p <0.05 from Mann Whitey U test. Data was log transformed and pareto scaled using Metaboanalyst 4.0. UP: upregulated; DOWN: downregulated.
